# Antibiotic Resistance and the Return to a Pre-Antibiotic Era: A Critical Narrative Review of a Global Catastrophe

**DOI:** 10.3390/biomedicines14092053

**Published:** 2026-09-12

**Authors:** Shaurya Prakash, Saloni Saini, Mahima Bharti, Saveg Yadav, Pravin Hivare, Neeraj Kumar Rai

**Affiliations:** 1 Department of Biochemistry, Central University of Haryana, Mahendragarh 123031, HR, India; shauryasinha097@gmail.com; 2 Department of Chemical Engineering, Indian Institute of Science Education and Research (IISER) Bhopal, Bhopal 462066, MP, India; 3 Huntsman Cancer Institute, University of Utah, Salt Lake City, UT 84112, USA; 4 Department of Neurology, University of Utah, Salt Lake City, UT 84112, USA; 5 Nora Eccles Harrison Cardiovascular Research Training Institute, University of Utah, Salt Lake City, UT 84112, USA

**Keywords:** antimicrobial resistance, silent pandemic, pre-antibiotic era, One Health, global health security, ESKAPE pathogens, antibiotic stewardship

## Abstract

Antimicrobial resistance is a growing global crisis that threatens to return humanity to a pre-antibiotic era where common infections become deadly. This narrative review synthesizes evidence from 2000 to early 2026, including Global Research on Antimicrobial Resistance data and World Health Organization surveillance, to outline the problem’s scale, drivers, and solutions. In 2019, bacterial resistance directly caused 1.27 million deaths and was linked to 4.95 million more. Low- and middle-income countries bear the heaviest burden. ESKAPE pathogens, especially carbapenem-resistant *Acinetobacter baumannii* and NDM-producing *Klebsiella pneumoniae*, drive intensive care unit mortality near 50% and cause untreatable neonatal sepsis. One Health drivers include antibiotic overuse in humans, with 30% of prescriptions unnecessary in high-income settings; agriculture, consuming 70% of global antibiotics; and environmental pollution, with resistance genes found in 72% of rivers. Bacteria spread resistance through horizontal gene transfer and mutations such as *gyrA* S83L, creating pan-drug-resistant strains that make surgeries, transplants, and cancer treatment risky. Economic modeling studies suggest that unchecked antimicrobial resistance could reduce annual global GDP by 1.1–3.8%, with some scenarios projecting losses of up to approximately 5% by 2050, depending on assumptions about healthcare costs, labor productivity, and livestock production. Solutions require subscription-based payment models, enforceable agricultural regulations, integrated genomic surveillance, and equity-focused diagnostics for low- and middle-income countries. Without binding 2030 targets, the post-antibiotic era would become a clinical reality within a decade.

## 1. Introduction

**Figure 1:**
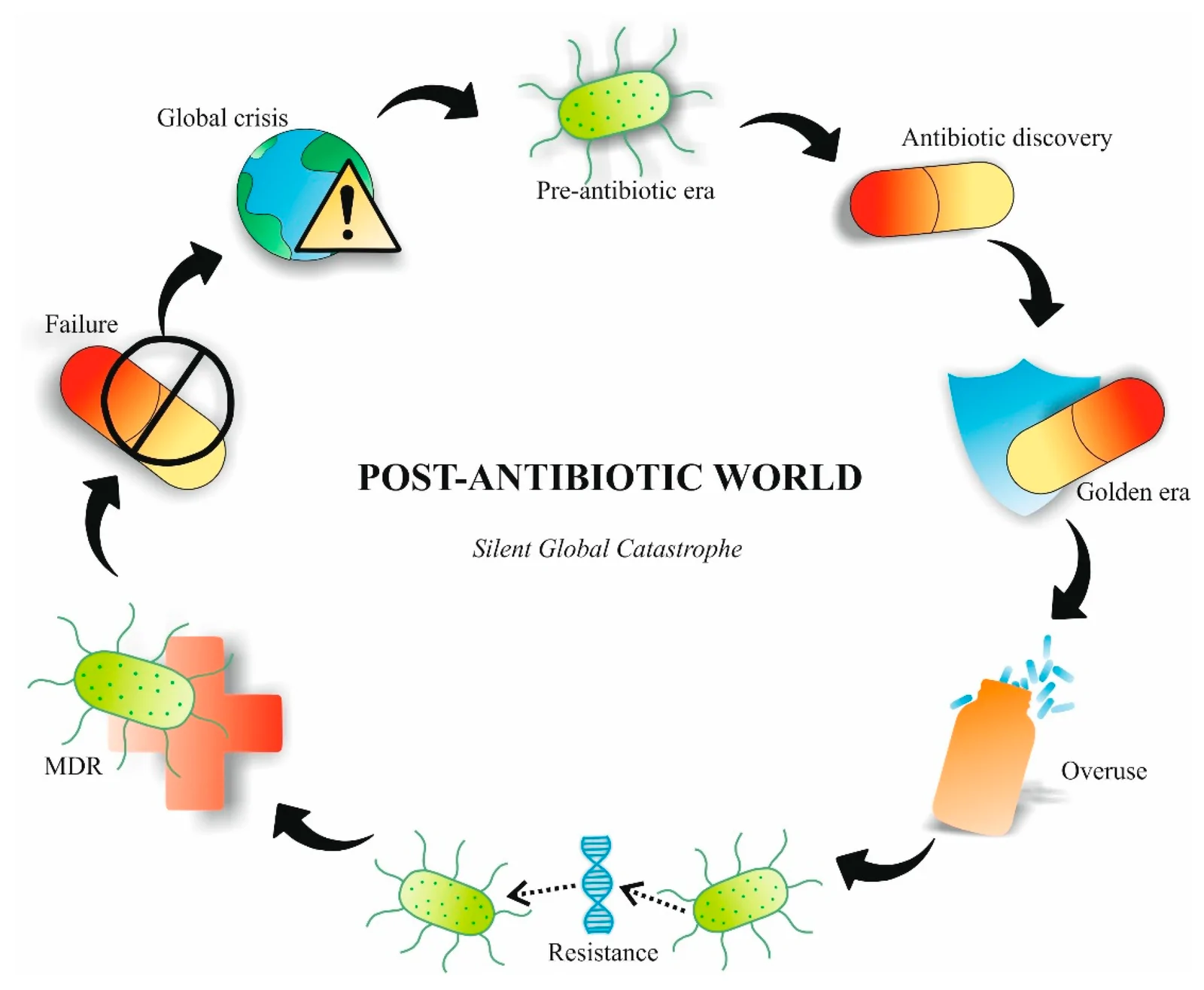
Conceptual illustration of the AMR cycle showing the transition from the pre-antibiotic era to the golden era of antibiotics, followed by overuse, resistance development, MDR, therapeutic failure, and the gradual return to a post-antibiotic world, representing a silent global health catastrophe.

In 1945, when Alexander Fleming accepted the Nobel Prize for the discovery of penicillin, he offered a prescient warning that would echo through the decades. He cautioned that the thoughtless person playing with penicillin treatment is morally responsible for the death of the man who succumbs to infection with the penicillin-resistant organism [1]. The discovery of penicillin in 1928 marked the beginning of the golden age of natural product antibiotic discovery, an era during which antibiotics dramatically changed modern medicine by reducing mortality from infectious diseases such as tuberculosis and pneumonia, and extending the average human lifespan by 23 years [2]. Penicillin and future antibiotics not only cured a wide range of bacterial infections but also created a scaffold to support medical innovations; routine and emergency surgeries were made safe, and cancer therapy and transplant medicine became possible. By the early 21st century, unregulated availability and widespread antibiotic use had made antimicrobial resistance (AMR) an everyday reality, threatening to unravel the very advances penicillin enabled. We now face a reality where the foundational drugs of modern medicine are failing at an alarming rate, threatening to undo a century of medical progress [3]. As illustrated in Figure 1, AMR follows a cyclical progression beginning with the pre-antibiotic era and the discovery of antibiotics, leading to the golden era of effective antimicrobial therapy. However, extensive and irrational antibiotic use creates strong selective pressure on bacterial populations, driving the emergence of resistant strains through genetic adaptation and horizontal gene transfer (HGT). This results in multidrug-resistant (MDR) pathogens, therapeutic failure, and an escalating global health crisis, ultimately pushing humanity toward a post-antibiotic world characterized by limited treatment options and increased mortality [4].

Antibiotics are not merely one class of pharmaceutical agents among many; they act as the essential infrastructure of modern healthcare [5]. Furthermore, advanced treatments for cancer, including chemotherapy and immunotherapy, rely heavily on the ability to prevent and treat opportunistic infections in immunocompromised patients [6]. Without urgent global action, the continued erosion of antibiotic effectiveness threatens to unwind a century of medical progress, returning humanity to a reality where common infections and routine procedures once again carry life-threatening risks [7].

Antibiotics were celebrated as revolutionary treatments, but their excessive use across human and veterinary medicine has significantly accelerated the development and dissemination of resistant bacterial strains [8]. However, AMR poses a major threat to many routine and advanced medical interventions that depend on the ability to prevent infection. The safety of common surgical procedures, the feasibility of organ transplants, and the administration of cancer therapies all depend on the efficacy of antibiotic drugs to manage and treat bacterial infections [9]. This self-reinforcing cycle of antibiotic overuse accelerates the evolution of bacterial defenses, threatening to return society to a pre-antibiotic era where the efficacy of essential medicines fails, and simple infections once again become fatal [10].

Unchecked antibiotic overuse has allowed bacteria to evolve into three increasingly threatening categories. Some are classified as MDR when they resist at least one agent in three or more antimicrobial classes. Others are labeled extensively drug-resistant (XDR) when they remain susceptible to only one or two classes. The most severe are pandrug-resistant (PDR) strains, which are non-susceptible to every agent in all tested antimicrobial categories [11]. Bacteria acquire and spread antibiotic resistance through three primary mechanisms: spontaneous genetic mutations, HGT of resistance genes between microbes, and uptake of resistance elements from the surrounding environment. Resistance depends on a variety of genetic factors, some of which can move between microorganisms through complex pathways. These processes have contributed to the emergence of major hospital-associated pathogens including methicillin-resistant *Staphylococcus aureus* (MRSA) and vancomycin-resistant *Enterococcus* (VRE) [12].

Generating the political will and societal urgency to address AMR remains a difficult task. Unlike a visible and rapidly spreading viral outbreak, AMR is an invisible crisis that tends to unfold beyond the immediate perception of the general public. As a result, the threat of untreatable infections often fails to galvanize the same immediate, high-level political reaction, even though its long-term consequences are catastrophic [13]. AMR is a silent pandemic driven by antibiotic overuse in humans and animals. It threatens global health systems, food security, and development. Earlier modeling studies projected that annual deaths attributable to AMR could rise from approximately 700,000 to 10 million by 2050 if no effective interventions were implemented. More recent empirical analyses, such as the GRAM 2019 study, estimate that 1.27 million deaths were directly attributable to bacterial AMR in 2019 alone, with 4.95 million deaths associated with resistance. Together, these data indicate that observed mortality is already within the range anticipated by earlier worst-case scenarios [14]. The molecular architecture of AMR is driven by active efflux, HGT, enzymatic degradation, membrane impermeability, and target modification [15]. Efforts to combat AMR are further hindered by a lack of public knowledge and misconceptions about its origins [16]. This has led researchers to stress that fostering global cooperation and prioritizing strategic antimicrobial use are as vital as developing new drugs, aiming to prevent a future where even simple bacterial infections could once again turn fatal [17].

This narrative review critically analyses the current trajectory of global AMR and examines the factors driving us toward this precipice. We argue that the transition to a pre-antibiotic era is no longer a theoretical risk for the distant future but is already an unfolding reality in specific geographic regions and clinical domains. In Eastern India, a multi-country study found mortality from neonatal sepsis remains high as pathogens routinely escape common empiric regimens, with 70% of affected newborns requiring escalation to higher-end antibiotics [18]. In a Northern Indian Neonatal Intensive Care Unit (NICU), an outbreak of colistin-resistant *K. pneumoniae* led to a case where no sensitive antibiotic was available for a septic newborn, solely based on available susceptibility data [19]. Meanwhile, in western sub-Saharan Africa, colistin, the last-line therapy, is already failing due to rising resistance driven by the *mcr-1* gene [20].

In response to the gaps highlighted by the COVID-19 pandemic, the 78th World Health Assembly adopted the Pandemic Agreement in May 2025 to enhance international coordination for pandemic prevention, preparedness, and response [21]. To effectively address these pressing challenges, sustained political commitment, innovative financing, and the greater integration of infectious disease strategies within broader health systems are urgently required [22]. The rise in AMR, coupled with the emergence of opportunistic infections in an increasingly immunocompromised population, has amplified the urgency for rapid, reliable, and accessible solutions [23]. This review aims to clarify the stakes of this silent catastrophe as it seeks to evaluate whether current global strategies are sufficient to prevent a permanent shift in the landscape of modern medicine or if a more radical rethinking of our relationship with microbes is required to avert a post-antibiotic future.

## 2. Review Methodology

This narrative review was designed to synthesize clinically, economically, and mechanistically relevant evidence on AMR and its implications for a potential return to a pre-antibiotic era. Relevant literature was identified through targeted searches of PubMed/MEDLINE, Scopus, Web of Science, and Google Scholar for peer-reviewed articles, major surveillance reports (including GRAM, WHO GLASS, CDC, and ECDC), and policy documents published between January 2000 and March 2026. Search terms included combinations of “antimicrobial resistance,” “post-antibiotic era,” “ESKAPE pathogens,” “One Health,” “antibiotic stewardship,” “phage therapy,” “antimicrobial peptides,” “photodynamic therapy,” “quorum sensing,” “antibiotic pipeline,” and “global AMR governance.” We prioritized systematic reviews, large observational studies, global and regional surveillance reports, and high-impact primary research. Articles were included if they provided quantitative data on AMR burden, mechanisms, drivers, alternative therapies, or policy and economic analyses relevant to high-income and low- and middle-income countries. Reviews and editorials were used selectively to contextualize findings. No formal quality scoring was applied; instead, emphasis was placed on studies with robust sample sizes, validated methods, and consistency across multiple sources. Data were synthesized narratively, with particular attention to convergence between independent datasets (e.g., GRAM mortality estimates, WHO surveillance, economic modeling studies).

## 3. The Unfolding Catastrophe: Clinical and Economic Burden

### 3.1. The Burden of Disease: Moving Beyond Estimates

For many years, the global health community relied on statistical models and extrapolations to gauge the threat of AMR. While these projections were alarming, they lacked the empirical weight to truly shift policy. This landscape changed fundamentally with the publication of the GRAM report in *The Lancet* in 2022 [24]. By aggregating data from 204 countries and territories, a landmark 2022 study revealed that in 2019 alone, 1.27 million deaths were directly attributable to bacterial AMR [24]. When accounting for deaths associated with resistance, where AMR played a contributing role, that number rose to 4.95 million [25].

The burden of AMR is profoundly unequal, representing not merely a biological phenomenon but a direct consequence of global socioeconomic disparities [26]. In Sub-Saharan Africa, the high mortality from *K. pneumoniae* infections, approximately 800,000 global deaths in 2019, with 80% associated with AMR, is compounded by a pooled prevalence of carbapenem-resistant *Acinetobacter baumannii* (CRAB) of 20% [27,28]. The global dominance of ESKAPE pathogens symbolizes the collapse of antibiotic reliability and reflects the structural failure of modern antimicrobial therapy, as summarized in Table 1 [29].

### 3.2. The End of Modern Medicine: The Prophylaxis Void

The concept of a pre-antibiotic era is often misunderstood as simply a time when active infections cannot be treated. However, a more profound and systemic threat lies in the potential loss of antibiotic prophylaxis, which is the preventative use of antibiotics that underpins much of modern medicine [37]. Antibiotics are essential to contemporary medicine, as they underpin invasive surgical procedures and immunosuppressive therapies that depend on effective infection prevention [37].

This threat is compounded by the emergence of PDR strains of bacteria that are impervious to all available agents, including last-resort drugs like colistin and cefiderocol [38]. The identification of the *mcr-1* gene, which confers plasmid-mediated resistance to colistin, in human isolates across multiple continents is a clear warning sign [39]. It signals the potential closure of the therapeutic window for complex medical interventions. Increasing rates of fluoroquinolone resistance have reduced the overall benefit of fluoroquinolone prophylaxis, resulting in a lack of demonstrable mortality benefit and diminished efficacy in preventing Gram-negative bloodstream infections [40]. This shift accelerates the development of carbapenem resistance, further narrowing the options for treating the most vulnerable patients and threatening the viability of aggressive cancer therapies.

### 3.3. Economic Fallout

The economic consequences of unchecked AMR are as severe as the clinical ones. A 2017 World Bank report estimated that by 2050, uncontrolled AMR could reduce annual global GDP by 1.1% to 3.8%, a level of economic shock comparable to the 2008 global financial crisis but sustained over decades rather than years. Subsequent modeling exercises, incorporating broader assumptions about healthcare expenditure, labor-force attrition, and livestock productivity, have projected upper-bound losses approaching 5% of global GDP under worst-case AMR trajectories. These higher estimates reflect more expansive cost categories and more severe resistance scenarios, but all models converge on the conclusion that AMR imposes macroeconomic risks of historic magnitude [41]. Yet even these dire forecasts likely understate the true cost, as conventional economic models struggle to fully account for the loss of human capital and the disintegration of healthcare efficiency.

A 2023 Italian study analyzed 174 healthcare-related septic episodes caused by CRAB, *K. pneumoniae*, and *Pseudomonas aeruginosa.* It reported a mean excess hospitalization of 19 days per patient and total excess expenditure of €3.19 million, with prolonged stays accounting for 85% of costs and antimicrobial therapy for 11% [42]. When these incremental costs are extrapolated globally, they represent an unsustainable financial burden. This is particularly true for health systems in LMICs, which are often already operating at their absolute limits and cannot absorb the additional costs of prolonged care and expensive second-line drugs.

## 4. The Engine of Resistance: A One Health Perspective

### 4.1. Human Consumption: Access vs. Excess

The primary driver of AMR in human health operates through a paradox defined by simultaneous excess and scarcity. This access-versus-excess dynamic creates a dual pressure on global resistance patterns. In high-income countries and rapidly emerging economies, the challenge is predominantly one of excess, where the overuse and misuse of antibiotics fuel resistance. Yet, in many LMICs, the problem is one of scarcity, where millions die from treatable infections simply because effective antibiotics are not available or affordable [43]. Antibiotics are frequently prescribed for viral infections, such as the common cold or influenza, where they offer less therapeutic benefit [44]. The Centers for Disease Control and Prevention estimates that up to 30% of antibiotic prescriptions in primary care are unnecessary, largely because antibiotics are prescribed for conditions such as viral respiratory infections [45].

Conversely, in many LMICs, the driver is often a desperate lack of access to high-quality, appropriate treatments. When optimal first-line antibiotics are unavailable or unaffordable, patients and providers resort to substandard alternatives or inappropriate drug choices. A comprehensive 2023 systematic review of 162 studies across 52 countries found that 63.4% of community pharmacies worldwide dispensed antibiotics without a prescription, often driven by patient demand, financial incentives, and weak regulations, while cost pressures in LMICs frequently lead to patients rationing doses, creating ideal conditions for resistance to emerge and spread globally [46].

### 4.2. The Agricultural Industrial Complex

On a global scale, the consumption of antibiotics by animals far outstrips that of humans, making agriculture a massive engine for resistance. The intensification of livestock production to meet the protein demands of a growing global population relies heavily on antimicrobials [47]. Globally, it is estimated that approximately 70% of all antibiotic consumption occurs in the agricultural sector, predominantly for growth promotion and metaphylaxis in food-producing animals [48]. These drugs are used not just for treating sick animals, but for metaphylaxis, which involves the mass medication of entire herds or flocks to prevent disease outbreaks, and in many regions for growth promotion [49]. This continuous, low-dose exposure exerts profound selection pressure on the microbiome of food animals [50]. The consequences of this practice were starkly illustrated by the emergence of the *mcr-1* gene, a plasmid-mediated mechanism conferring resistance to colistin. Colistin is a crucial last-resort polymyxin drug for treating MDR Gram-negative infections in humans, yet its widespread use in pig farming facilitated the development of resistance that has since circumnavigated the globe [51].

The role of poultry as a reservoir for critically important carbapenemase and colistin resistance genes has been substantiated by studies employing advanced genomic methods. A 2025 study using whole-genome sequencing of 119 isolates from commercial chicken farms in China identified the widespread presence of both *bla*NDM carbapenemase genes and *mcr* colistin resistance genes [52]. Notably, the study found frequent co-occurrence of *bla*_NDM_ and *mcr* genes in 28 isolates, including combinations such as *bla*_NDM-5_/*mcr-1*, *bla*_NDM-5_/*mcr-3*, *bla*_NDM-5_/*mcr-8*, and *bla*_NDM-5_/*mcr-9,* demonstrating that these resistance determinants can reside together in the same bacterial strains. These findings reveal that chicken farms are significant reservoirs for both *bla*_NDM_ and *mcr* genes [52]. Similarly, research from live poultry markets in China detected *bla*_NDM_-positive strains, primarily in *Escherichia coli (E. coli)* and *K. pneumoniae*, and documented the coexistence of *bla*_NDM-5_ and *mcr-1* in five *E. coli* strains [53]. This evidence collectively underscores the role of the poultry production chain as a reservoir for high-priority carbapenemase and colistin resistance genes, highlighting a critical public health risk.

### 4.3. The Environmental Reservoir

The environment serves as the silent, often overlooked third pillar of the One Health framework, acting as both a reservoir for resistance genes and a mixing vessel where new combinations can emerge [48]. Surveillance studies have detected antibiotic-resistance genes in up to 72% of river water samples in some regions, highlighting the pervasive contamination of freshwater ecosystems by AMR determinants [54]. Pharmaceutical manufacturing hubs have historically discharged effluent containing extremely high concentrations of active pharmaceutical ingredients directly into local waterways [55,56]. The scale of this pollution is staggering; water samples taken from the Isakavagu stream near Hyderabad, India, a major center for generic drug production, revealed antibiotic concentrations exceeding 31 milligrams per liter [57]. These levels are thousands of times higher than therapeutic plasma levels found in treated patients, effectively turning local ecosystems into vast selection vessels for drug-resistant bacteria [55].

Environmental pollution plays a central role in AMR by creating selection hotspots such as manufacturing effluents, agricultural waste, and untreated sewage, which amplify resistance evolution and interspecies gene transfer via HGT mechanisms [57]. Hospital effluent is a recognized source of pharmaceutical residues and AMR determinants that survive conventional treatment, while agricultural runoff and household wastewater continuously seed soils and water with resistant bacteria and residual drugs [57,58]. Wastewater treatment plants are now recognized as critical pollution hotspots where selective pressure from residual antibiotics, heavy metals, and microplastics facilitates the proliferation of antimicrobial resistance genes (ARGs) [59]. India’s sewage systems are established reservoirs of ARGs, within which genetic exchanges among bacterial communities accelerate the evolution of AMR pathogens, posing dual threats to public health in community ecosystems and healthcare facilities [60].

The following Table 2 summarizes the effect of the triad of antimicrobial resistance, their role as the drivers and involvement in the transmission of resistance.

## 5. Molecular Resilience and Cellular Survival Programming

### 5.1. Genetic Origins of Resistance

**Figure 2:**
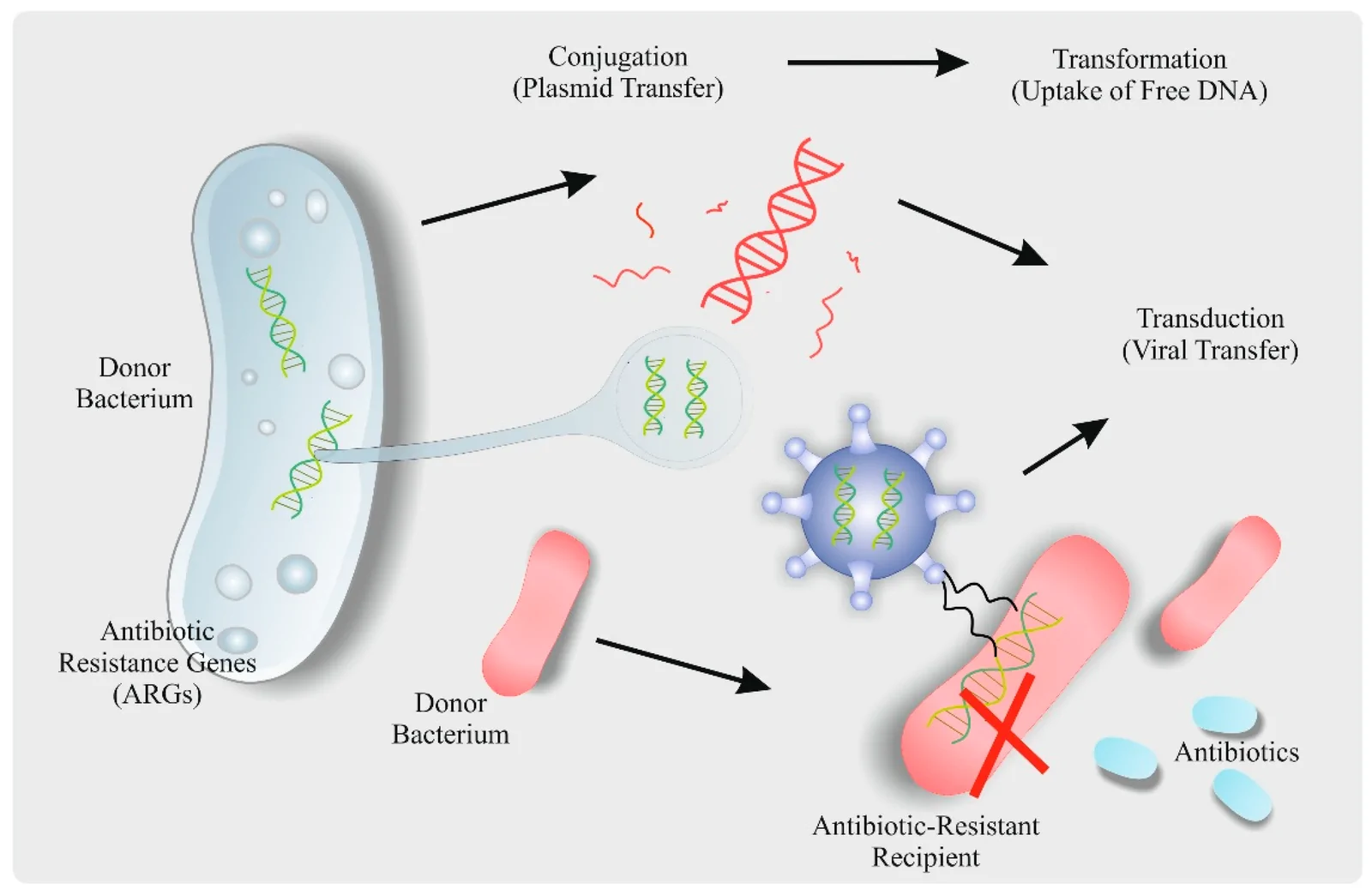
HGT mechanisms involved in the spread of antimicrobial resistance by conjugation, transformation, and transduction, leading to the emergence of AMR.

The genetic origins of AMR lie in spontaneous mutations and HGT, both of which are amplified by antibiotic selective pressure. Within-patient AMR emergence is driven by four principal mechanisms: spontaneous resistance mutations, in situ HGT of resistance genes, selection of pre-existing resistance, and immigration of resistant lineages [65]. Mobile genetic elements (MGEs) such as plasmids, transposons, and integrons are essential for facilitating HGT, carrying resistance genes across bacterial populations, and evolutionary competition [66]. Mutations in the quinolone resistance-determining regions (QRDRs) of the target genes *gyrA* and *parC* represent a primary de novo mechanism of high-level fluoroquinolone resistance in clinically relevant pathogens [67]. Under antimicrobial selection pressure, specific single-nucleotide changes, such as S83L and D87N in GyrA, or S80I and E84V in ParC, significantly reduce drug binding affinity, elevating minimum inhibitory concentrations (MICs) and driving clinical treatment failures [67,68]. Studies have confirmed a positive correlation between the number and type of QRDR mutations and the degree of resistance, with double mutations in both *gyrA* and *parC* conferring the highest levels of resistance [69]. De novo chromosomal mutations can raise the MICs many-fold; these mutations are often accompanied by additional variants that either refine resistance or compensate for the initial fitness costs [70]. As shown in Figure 2, AMR spreads through three principal HGT mechanisms: conjugation, involving plasmid-mediated cell-to-cell transfer; transformation, through uptake of extracellular DNA; and transduction, via bacteriophage-mediated gene transfer. Together, these processes enable rapid acquisition of resistance determinants and promote the emergence of antibiotic-resistant bacterial populations under selective antibiotic pressure.

HGT serves as the dominant inter- and intra-species disseminator of pre-formed resistance determinants, mobilizing ARGs via MGEs like plasmids, transposons, and integrons across One health reservoirs; the three core mechanisms include transformation, transduction, and conjugation [71]. Microenvironments like biofilms serve as critical hotspots for the horizontal transfer of ARGs. This process is facilitated by close cell-to-cell contact and the presence of extracellular polymeric substances (EPS), which can be significantly enhanced by antimicrobial agents. Recent mathematical modeling has demonstrated that contaminants like toxic metals and antibiotics promote plasmid persistence by stimulating conjugation within biofilm structures [72]. In parallel, pathogen evolution studies using phylogenetic and metagenomic analyses demonstrate that HGT often outpaces de novo mutation in global AMR epidemics [70]. Environmental resistomes, particularly in wastewater, soils, and animal production systems, act as critical reservoirs that seed clinical pathogens via integron-mediated capture of novel carbapenemases and other resistance determinants [73]. These synergistic dynamic mutations seeding novelty, HGT amplifying dissemination, underscore genomic complexity, including regulatory interplay and fitness trade-offs, demanding integrated stewardship, phage therapy, and pan-genomic prediction models to curb the escalating crisis.

### 5.2. Molecular Interference with Drug-Target Interactions

**Figure 3:**
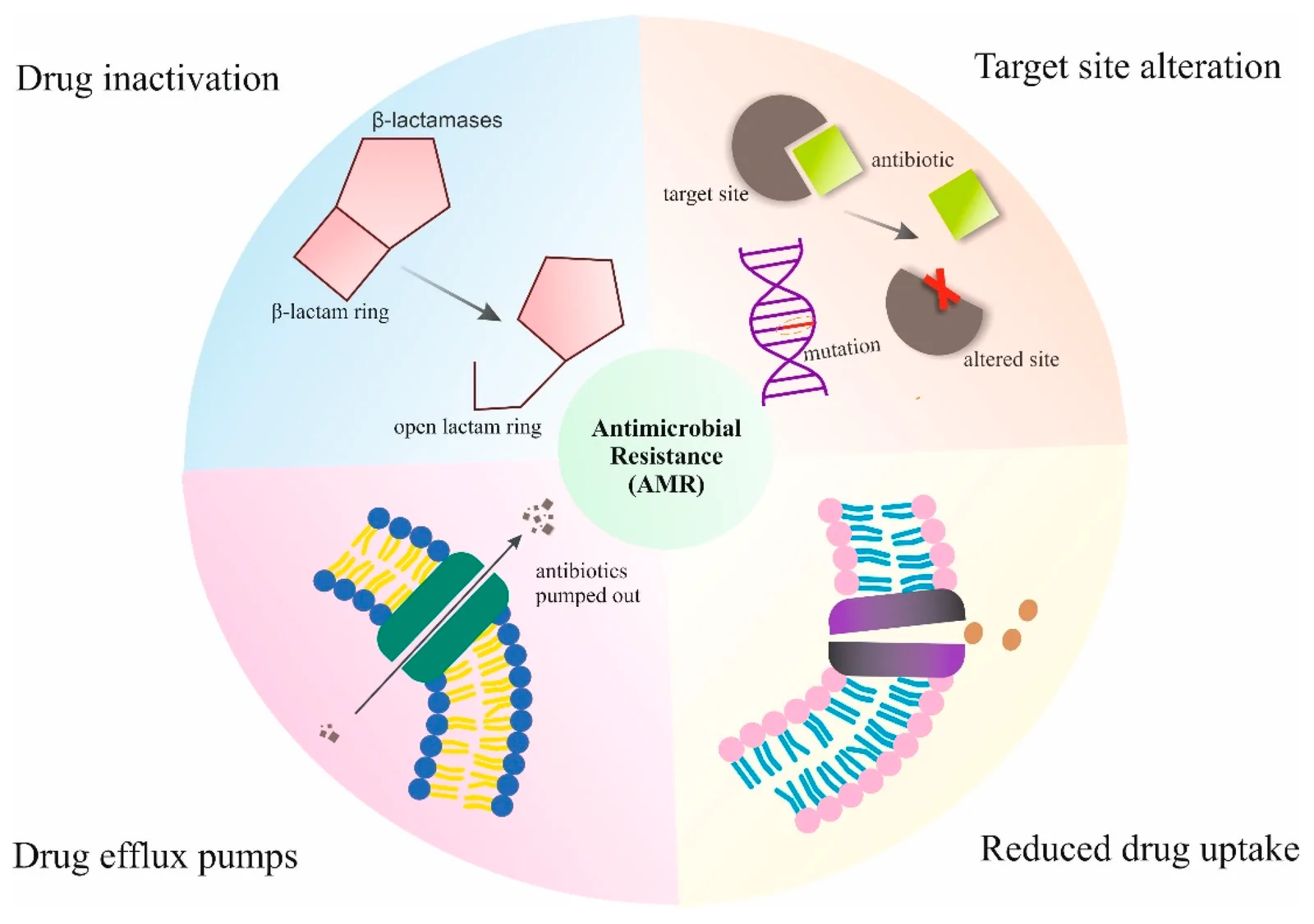
Membrane-associated AMR mechanisms showing active drug efflux and reduced membrane permeability through porin loss or altered uptake channels, collectively lowering intracellular antibiotic concentrations and promoting multidrug resistance.

The molecular mechanisms of AMR translate genetic mutations into functional phenotypes that shield pathogens from antibiotic lethality through four main categories. Enzymatic drug inactivation, such as the hydrolysis of β-lactam antibiotics by β-lactamases [74]. Target site alteration, including point mutations in the QRDRs of *gyrA* and *parC*, reduces drug binding and can elevate MICs many-fold [75]. Reduced drug accumulation results from decreased membrane permeability or overexpression of multidrug efflux pumps such as MexAB-OprM in *P. aeruginosa* [76,77]. Finally, metabolic bypass pathways allow bacteria to circumvent a drug-blocked reaction by using alternative enzymes or rewired metabolic routes that are not susceptible to the inhibitor [78,79]. These adaptive strategies allow bacteria to survive and proliferate under antibiotic pressure, as illustrated in Figure 3.

Enzymatic inactivation neutralizes antibiotics directly through hydrolysis or acylation, making them unable to bind their target and disrupt cell wall synthesis [80]. Extended-spectrum β-lactamases like CTX-M and serine carbapenemases such as KPC-2, KPC-3, and OXA-48 all rely on an active-site serine residue for their function [81]. Specifically, CTX-M-15 and KPC enzymes hydrolyze the β-lactam ring, employing their active-site serine to break down the antibiotic’s core structure [82]. In contrast, class D enzymes like OXA-48 degrade carbapenems through a related acylation mechanism, also involving their active-site serine [83]. Metallo-beta-lactamases (MBLEs), including NDM-1 and VIM, use zinc ions to catalyze hydrolysis, effectively abolishing activity across broad-spectrum beta-lactams [84]. Enzymatic inactivation neutralizes antibiotics through chemical modification. Aminoglycoside-modifying enzymes (AMEs) are the primary resistance mechanism for this drug class. AAC(3)-II, an acetyltransferase, inactivates gentamicin and tobramycin by catalyzing the addition of an acetyl group to the antibiotic’s central ring [85]. Similarly, APH(3’)-Ia, a phosphotransferase, confers resistance by transferring a phosphate moiety from ATP to the 3’-hydroxyl of the aminoglycoside [86]. Chloramphenicol resistance is largely mediated by chloramphenicol acetyltransferase (CAT), which catalyzes the acetyl-S-CoA-dependent acetylation of chloramphenicol at the 3-hydroxyl group, producing a modified drug that can no longer bind to ribosomes [87].

Target site alterations diminish antibiotic binding affinity through point mutations, acquired genes, or protective proteins that reshape critical interaction sites [88]. Ribosomal methylases such as ErmB dimethylate the A2058 position in 23S rRNA, blocking macrolide access to the peptidyl transferase center [89]. Tetracycline resistance involves proteins like TetO and TetM that protect the ribosome’s A-site [89]. In MRSA, the mecA gene encodes penicillin-binding protein 2a with low-affinity allosteric sites that sustain cell wall transpeptidation despite beta-lactam presence [90]. Quinolone resistance arises from gyrA and parC mutations like S83L or S80F, which distort DNA gyrase and topoisomerase IV binding pockets [91]. Rifampicin resistance in *Mycobacterium tuberculosis (M. tuberculosis)* is primarily caused by mutations in the *rpoB* gene, with the H526Y substitution being a clinically significant variant. At a molecular level, this mutation alters the structure of the RNA polymerase rifampicin-binding pocket, creating a steric hindrance that physically blocks the antibiotic from binding [92].

Resistance-nodulation-division family pumps, such as AcrAB-TolC in *Enterobacteriaceae* or MexAB-OprM in *P. aeruginosa*, form tripartite complexes powered by proton motive force to eject beta-lactams, quinolones, tetracyclines, and more [93]. The efflux of the antifungal agent Echinocandin B via the ATP-binding cassette (ABC) transporter EcdLp in *Aspergillus nidulans* highlights how microbial transporters can facilitate the secretion of self-produced bioactive molecules [94]. Major facilitator superfamily pumps like NorA and ATP-binding cassette transporters provide additional efflux capacity [95]. Permeability reductions further strengthen resistance by deletion of the OprD porin in *P. aeruginosa*, which limits carbapenem influx [96]. In many opportunistic pathogens, these systems operate in concert to establish multi-layered resistance phenotypes. Resistance-nodulation-division (RND) tripartite pumps such as AcrAB-TolC and MexAB-OprM span the inner membrane, periplasm, and outer membrane, enabling direct extrusion of diverse antibiotics from the cytoplasm or periplasm to the extracellular space [97]. Major facilitator superfamily (MFS) pumps and ABC transporters provide additional capacity, with ABC systems coupling ATP hydrolysis to substrate efflux and often contributing to high-level resistance when overexpressed [8,97].

Metabolic bypass pathways evade antibiotics by providing redundant or alternative routes around inhibited essential processes. Sulfonamide resistance illustrates this through mutations in dihydropteroate synthase that alter the para-aminobenzoic acid binding pocket, or plasmid-encoded sul1, sul2, and sul3 genes, producing insensitive enzyme variants to restore folate biosynthesis [98]. Plant-derived extracts exert antibacterial effects by interfering with multiple drug-target interactions, including disruption of cell wall synthesis, inhibition of key metabolic enzymes, and suppression of efflux pumps and quorum-sensing pathways [99]. Structural studies reveal conserved catalytic triads in these enzymes, underscoring their evolutionary robustness. Similarly, VRE circumvent antibiotic activity by acquiring *van* operons such as *vanA* and *vanB*, which lead to the production of modified cell wall precursors, D-Ala-D-Lac that vancomycin cannot bind effectively [100].

These interconnected mechanisms frequently co-occur in multidrug-resistant strains, amplifying selective pressure and complicating treatment. Their plasmid-mediated mobility, combined with biofilm persistence and persister cell formation, demands innovative countermeasures like combination therapies, phage therapy, and photodynamic approaches to disrupt this evolutionary arms race. Recent genomic surveillance highlights global dissemination, urging accelerated development of resistance-breaking agents.

### 5.3. Dynamic Cellular Reprogramming Under Antimicrobial Pressure

Biofilm formation represents a key non-genetic survival strategy that enables bacteria to withstand antibiotic treatment through multiple coordinated mechanisms. The self-produced EPS matrix composed of polysaccharides, proteins, and eDNA acts as a physical diffusion barrier that restricts antimicrobial penetration [101]. Within the biofilm, the formation of oxygen and nutrient gradients induces metabolic heterogeneity, with deeper layers exhibiting reduced metabolic activity that downregulates the very targets of many antibiotics, such as cell wall synthesis enzymes and ribosomes [102]. Quorum-sensing (QS) systems further regulate matrix production, virulence factor expression, and community dispersal, thereby enhancing collective tolerance [103]. Consequently, biofilm-embedded cells can exhibit 10- to 1000-fold higher tolerance to antimicrobial agents compared to their planktonic counterparts [104]. Together, these interconnected mechanisms create a protected niche where bacterial persistence, tolerance, and resistance evolution are synergistically amplified. Persister cells emerge as a transient, non-inherited subpopulation achieving metabolic dormancy through growth arrest and low ATP states, evading bactericidal action of cell wall inhibitors, protein synthesis blockers, or DNA-damaging agents without genotypic changes [105]. Toxin-antitoxin (TA) modules like HipA or RelE enforce this reversible tolerance, distinguishing it from resistance and fueling chronic infection relapses as seen in biphasic kill curves of *M. tuberculosis* [106]. Overarching these mechanisms are stress response pathways that sense sublethal antibiotic exposures and trigger protective cascades. The SOS regulon activates error-prone DNA polymerases such as PolV to promote mutagenesis while simultaneously inducing filamentation [107]. Oxidative stress systems upregulate superoxide dismutases and catalases to neutralize reactive oxygen species (ROS) generated by aminoglycosides or β-lactams [108]. Heat-shock chaperones, particularly those within the Hsp90-Hsp70 chaperone network, maintain proteostasis by refolding drug-induced protein aggregates and preventing further misfolding [109]. Concurrently, Gram-negative bacteria deploy envelope stress responses as a frontline defense. The two-component system PmrAB is a key sensor that remodels the bacterial surface by modifying lipid A with cationic phosphoethanolamine or 4-amino-L-arabinose, directly neutralizing the electrostatic interaction with colistin and conferring resistance [110]. These pathways amplify multidrug survival and bridge transient tolerance to heritable resistance in clinical settings.

It is essential to distinguish between resistance, tolerance, and persistence, as these phenotypes have different implications for treatment and drug development. Resistance refers to heritable genetic changes that increase the MIC required to inhibit growth, enabling bacteria to proliferate at drug concentrations that would normally be lethal [111]. Tolerance describes a reversible, phenotypic ability to survive transient exposure to high antibiotic concentrations without a change in MIC, often through slowed growth or metabolic dormancy [111]. Persistence is a subset of tolerance in which a small subpopulation of cells enters a dormant or slow-growing state, surviving lethal antibiotic exposure and subsequently repopulating once treatment ceases [112]. While resistance is detected by standard susceptibility testing, tolerance and persistence require time-kill assays and specialized protocols. Therapeutically, resistance demands alternative agents or combinations, whereas tolerance and persistence may be countered by drugs that disrupt dormancy, biofilm matrices, or stress-response pathways, or by optimizing dosing schedules to eradicate slow-growing subpopulations [111].

### 5.4. Systems-Level Adaptation Driven by Biological Regulation

Systems-level resistance biology elucidates how AMR transitions from individual cellular adaptations to population-wide dominance, embodying a dynamic balance between survival advantages and inherent biological fitness costs, continually reshaped by environmental and therapeutic selective pressures. Quinolone resistance often arises from the *gyrA* S83L mutation, which alters DNA gyrase topology, reducing drug binding but slowing replication fork progression and imposing a measurable fitness cost [113]. Similarly, streptomycin resistance mediated by the *rpsL* K42N mutation modifies the ribosomal S12 protein, impairing translational fidelity and reducing growth rates [113]. This compensatory effect enables stable persistence of resistant bacteria within antibiotic-free niches, such as commensal microbiomes. This interplay fuels adaptive evolution, where hypermutable strains accelerate resistance trajectories, fostering clonal expansion of MDR lineages like *K. pneumoniae* ST258 through within-host diversification and inter-host transmission chains [114]. Biofilms and persister reservoirs act as evolutionary innovators, generating genetic diversity through HGT and SOS-induced mutagenesis, which accelerates the development of antibiotic resistance [115]. Thus, resistance evolution transcends single cells, manifesting as predictable population-level adaptations demanding stewardship that anticipates compensatory trajectories and transmission networks.

## 6. Alternative Antimicrobial Strategies in the Post-Antibiotic Era

### 6.1. Enzyme- and Phage-Based Antimicrobial Therapies

**Figure 4:**
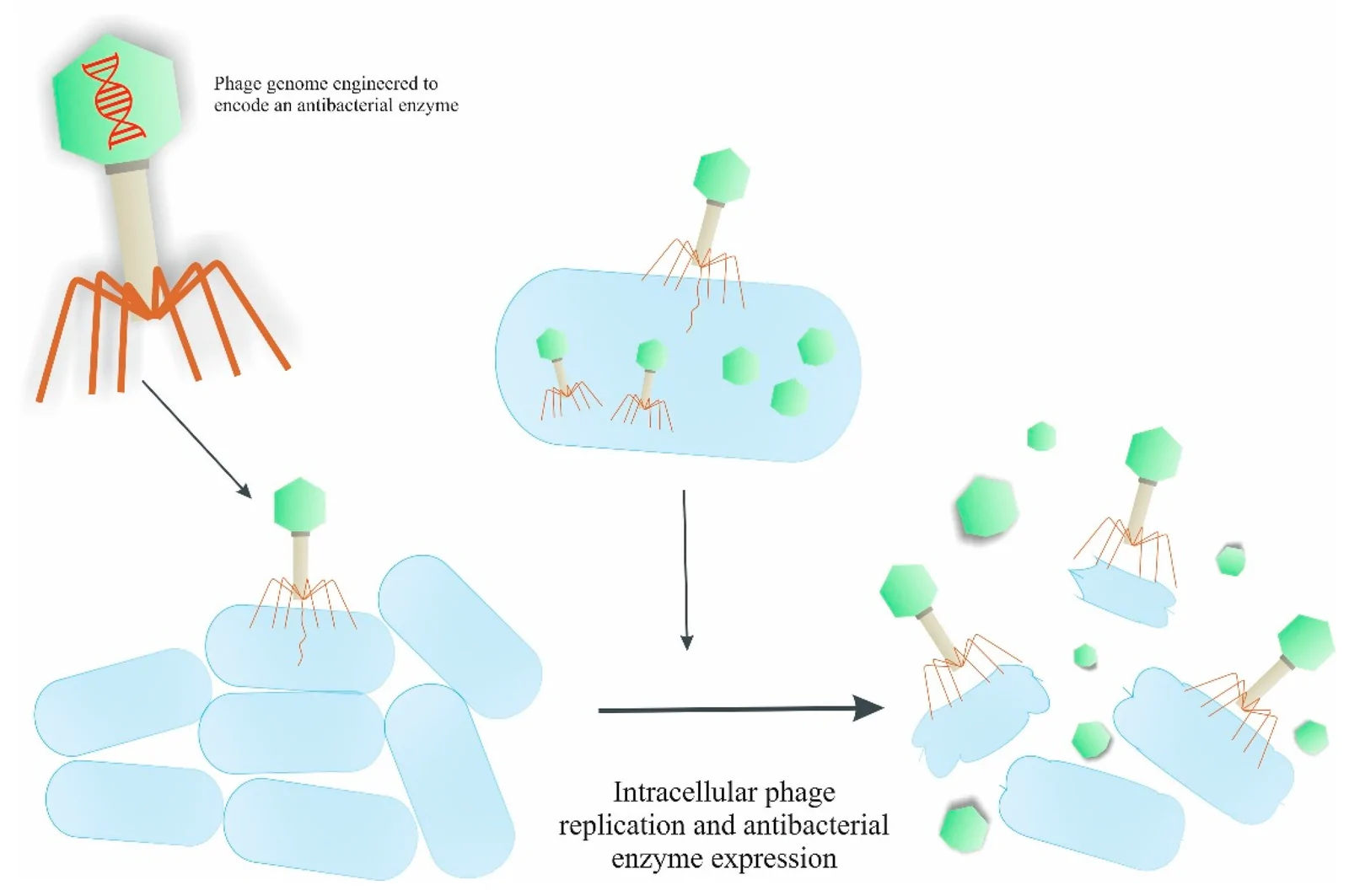
Schematic representation of enzyme-armed bacteriophage therapy. Engineered bacteriophages deliver genes encoding antibacterial enzymes into target bacteria, where intracellular replication leads to simultaneous progeny phage synthesis and enzyme expression. Upon host lysis, released phages and enzymes cooperatively eliminate neighboring bacterial cells, enhancing antibacterial efficacy against resistant populations.

Enzyme- and phage-based therapies are designed to dismantle these resistance barriers by exploiting structural vulnerabilities in bacteria; however, clinical translation is constrained by narrow host range, immunogenicity, delivery barriers, stability problems, manufacturing complexity, and regulatory uncertainty [116]. They target the peptidoglycan sacculus, a single, continuous macromolecular scaffold that surrounds the cytoplasmic membrane and maintains structural integrity under substantial osmotic stress, with recent studies emphasizing its role in coupling with the outer membrane to resist lysis during turgor-driven mechanical loads in Gram-negative bacteria [116]. They also attack polysaccharide capsules that shield Gram-negative bacteria from immune recognition and phagocytosis; recent work highlights that these capsules modulate innate immune responses, impair opsonophagocytosis, and can even alter the local inflammatory environment in the gut, thereby enhancing bacterial persistence and transmissibility [116]. In chronic infections, much of the bacterial population resides within biofilms, where extracellular DNA, proteins, and exopolysaccharides such as poly-β-1,6-N-acetyl-D-glucosamine or alginate form a dense matrix [117]. This matrix severely restricts antibiotic diffusion, mimicking a medieval fortress, and enzyme or phage strategies aim to degrade these scaffolds to restore drug access. As depicted in Figure 4, engineered bacteriophages deliver genes encoding antibacterial enzymes into target bacterial cells. Following infection, the phage replicates intracellularly and drives expression of these enzymes, leading to effective bacterial lysis. By degrading capsular polysaccharides and peptidoglycan, these enzymes compromise the structural integrity of the cell envelope at sites of high turgor pressure. In Gram-negative bacteria, even localized weakening of the peptidoglycan layer can lead to osmotic lysis, as the inner membrane is no longer supported against the cell’s internal osmotic pressure [118]. This mechanism underlies the rapid bactericidal activity of many phage-encoded endolysins and depolymerases when delivered directly to the periplasm or cell surface. This approach enhances phage-mediated killing and provides a targeted strategy against resistant bacterial populations, but a narrow host range, bacterial escape, and immune clearance can limit durability and broad clinical deployment. Non-traditional antibacterial agents encompass diverse modalities, including bacteriophages, lysins, antibodies, microbiome-based approaches, nucleic acids, and anti-toxin strategies, but their development is constrained by recurring hurdles such as dose selection, patient enrollment, limited validated surrogate endpoints, and the need to demonstrate clinically meaningful benefit under existing regulatory standards [119].

Adsorption in tailed phages is mediated by receptor-binding proteins containing conserved N-terminal and C-terminal domains for host recognition [120]. For efficient DNA delivery, tail-associated enzymes degrade surface barriers; *Klebsiella* phage KP32 encodes two capsule depolymerases, KP32gp37 and KP32gp38, with strict specificity for capsular serotypes K3 and K21 [120]. Peptidoglycan penetration is facilitated by virion-associated lysozyme domains that cleave glycosidic bonds, which in phage T7 is essential for successful infection. Once inside, phage components also reshape the host, as demonstrated by the endolysin Ply500 from *Listeria* phage A500, its cell wall binding domain (CBD500) recognizes 3’-O-acetylated GlcNAc residues in wall teichoic acid, ensuring selective lysis [120]. The colanidase tailspike protein gp150 of *Escherichia* phage Phi92 hydrolyzes the exopolysaccharide colanic acid with high specificity. X-ray crystallography at 1.80 Å resolution revealed a hydrolase structure co-crystallized with a colanic acid repeating unit, detailing the substrate-binding pocket [121]. For phage- and lysin-based therapies, the main translational barriers include narrow host range, immunogenicity, manufacturing complexity, regulatory uncertainty, and the challenge of demonstrating benefit in acute infection settings where rapid treatment decisions are required [119,122].

Based on the comprehensive review, a diverse arsenal of enzyme-based therapeutics is emerging to combat multidrug-resistant pathogens; nevertheless, their translation remains limited by rapid clearance, proteolytic degradation, tissue-penetration challenges, and scale-up constraints. Engineered endolysins (Artilysins) can rapidly hydrolyze the peptidoglycan layer of Gram-negative bacteria, achieving a 4 to 5-log reduction within 30 min, while lysostaphin specifically cleaves the pentaglycine cross-bridge of Staphylococcus aureus (*S. aureus*) peptidoglycan [123,124]. Alginate lyase SG4+ purified from Paenibacillus lautus has a half-life of 180 min at 37 °C and, when combined with gentamicin, eradicates 48.4–52.3% of mucoid P. aeruginosa biofilms; with amikacin, eradication reaches 58–64.6% [125]. An important cautionary lesson for the field is the failure of Contrafect’s lysin program, which illustrates how promising biological activity can still be derailed by formulation, development, and commercialization barriers [126].

Although enzyme therapies such as endolysins and depolymerases exhibit potent bactericidal activity in vitro, their in vivo application faces immunological and pharmacological barriers. Repeated administration may elicit neutralizing antibodies that reduce efficacy or cause hypersensitivity reactions. Systemic delivery is further complicated by rapid renal clearance, proteolytic degradation, and limited penetration into deep tissues and biofilms [127]. Promising formulation strategies include PEGylation to extend half-life, encapsulation in nanoparticles or liposomes, and local delivery via inhalation, catheters, or wound dressings. Engineering enzymes to reduce immunogenic epitopes and to enhance stability under physiological conditions represents an active area of research aimed at overcoming these translational hurdles [128]. Table 3 comprehensively discusses the mechanistic and therapeutic attributes of the enzyme- and phage-based therapies.

### 6.2. Antimicrobial Peptides (Amps)

The broader literature also shows that the value of these products depends on how they are categorized: some fit a Standalone, Transform, Augment, or Restore model, while others are designed primarily for population-level benefits such as reducing colonization or transmission [119,122]. Antimicrobial peptides (AMPs) are evolutionarily conserved innate immune effectors that have persisted for hundreds of millions of years of coevolution with microbes, retaining broad-spectrum efficacy against bacteria, fungi, protozoa, and viruses. Given the escalating challenge of antibiotic-resistant strains, AMPs offer a promising pathway for developing novel antimicrobial agents, although clinical translation has been slowed by toxicity, instability, and delivery challenges [141]. AMPs are short cationic peptides that typically adopt amphipathic α-helical or β-sheet conformations or display non-αβ elements. Their amphipathicity and net positive charge enable selective electrostatic interaction with negatively charged bacterial membranes, leading to membrane disruption through pore-forming models such as barrel-stave, toroidal, or carpet mechanisms without targeting intracellular components, thereby bypassing conventional resistance pathways and reducing the likelihood of resistance development [142]. Despite this promise, AMP development faces major barriers, including protease degradation, host cytotoxicity, high production costs, formulation instability, and limited in vivo pharmacokinetic durability. Products with diffuse public-health value may therefore require different development and reimbursement thinking than conventional antibiotics, because their principal benefits may accrue to future patients or to the wider population rather than to the treated individual alone [119].

Classically, polymyxin-type AMPs cyclize fatty acyl moieties to anchor to lipid-A phosphate groups, displacing Mg^2+^ bridges and disorganizing the outer membrane of Gram-negative bacteria, whereas lantibiotics such as nisin A/B wedge into lipid-II pyrophosphate, blocking transglycosylation and forming pores selectively in Gram-positive species where the pyrophosphate is accessible [143,144]. Unlike conventional pore-forming AMPs, daptomycin employs a distinctive calcium-dependent mechanism: it rapidly and reversibly binds phospholipid membranes in milliseconds, then proceeds to a slow, irreversible insertion step that requires the bacterial-specific lipid phosphatidylglycerol (PG) [145]. Fluorometric titration has shown that daptomycin simultaneously binds two PG molecules with nanomolar affinity in the presence of Ca^2+^, forming a stable 1:2 complex that facilitates selective membrane insertion and disruption [145].

Bacteriocins and microbiota-derived agents offer a further non-traditional avenue against multidrug-resistant Gram-negative pathogens. A bacteriocin purified from Lactobacillus acidophilus showed antagonistic activity against Klebsiella pneumoniae isolated from urinary tract infections [146], while a sterile microbiota-derived growth medium exhibited antagonistic and antibiofilm effects against *K. pneumoniae* in vitro [147]. As summarized in Table 4, these narrow-spectrum, microbiome-compatible strategies complement the enzyme- and peptide-based approaches already discussed, offering low collateral disruption of commensal flora and warranting further evaluation as antibiotic adjuncts within the post-antibiotic strategic landscape described earlier.

### 6.3. Photodynamic Therapy (Pdt) for Infection Control

**Figure 5:**
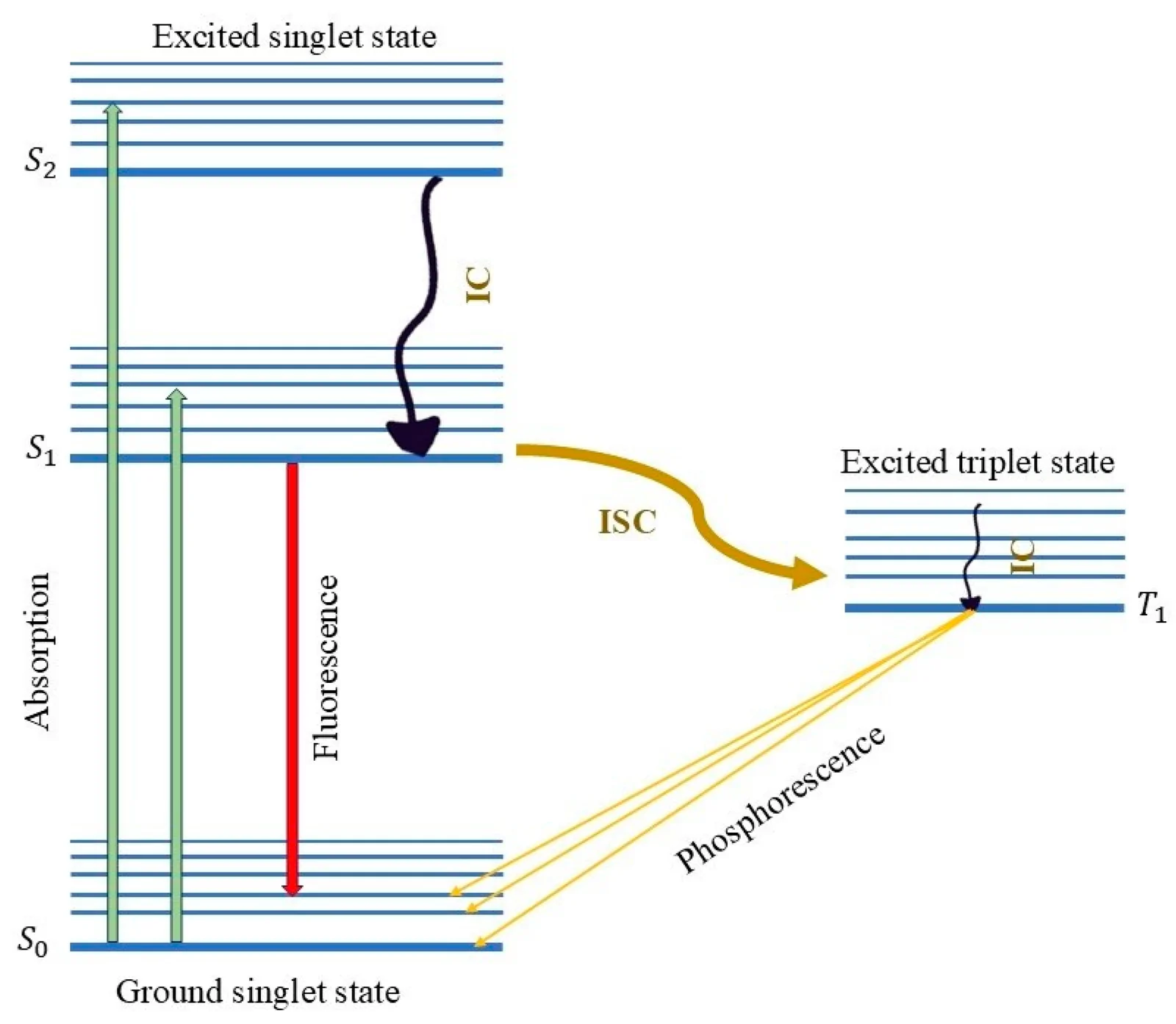
Jablonski diagram illustrating the photophysical transitions involved in antimicrobial photodynamic therapy. Upon light absorption, the photosensitizer transitions from the ground singlet state (S_0_) to an excited singlet state (S_1_), followed by fluorescence emission or intersystem crossing to the long-lived triplet state (T1). The triplet state drives phosphorescence and interacts with molecular oxygen to generate reactive oxygen species (ROS) through Type I and Type II pathways, leading to oxidative damage of bacterial membranes, proteins, nucleic acids, and biofilm matrices.

Photodynamic therapy harnesses a precise photophysical triad: a photosensitizer, a specific activating wavelength of light, and molecular oxygen to generate cytotoxic ROS for targeted cell destruction [160]. A key to its success is the use of red or near-infrared light, typically in the 600–850 nm range, which provides greater tissue penetration than shorter wavelengths [161]. This non-thermal, resistance-proof approach circumvents enzymatic efflux and target modification. The Jablonski framework forms the core photophysical basis for antimicrobial photodynamic therapy, as shown in Figure 5. However, photodynamic therapy is limited by light penetration depth, the need for local delivery, photosensitizer solubility issues, and variable efficacy in deep or poorly accessible infections.

When irradiated at the appropriate wavelength, the photosensitizer absorbs photons and rises from its ground singlet state to an excited singlet state [162]. Upon irradiation, a photosensitizer absorbs photons and transitions from its ground singlet state (S_0_) to an excited singlet state (S_1_). A portion of this energy is released as fluorescence, but most undergoes intersystem crossing to a stable, long-lived triplet excited state. This triplet state drives antimicrobial action by transferring energy or electrons to nearby molecular oxygen, generating powerful ROS, including singlet oxygen. These ROS inflict rapid, nonspecific oxidative damage on bacterial membranes, proteins, nucleic acids, and biofilm EPS, leading to irreversible cell death with a negligible risk of inducing drug resistance, as their multitarget oxidative action leaves little opportunity for adaptive evolutionary pathways [163,164]. ROS generation is not limited to photodynamic therapy; silver nanoparticles synthesized from *Bacillus subtilis* exhibit potent ROS-mediated staphylocidal activity against MRSA, achieving a MIC of 0.4 mg/mL and inhibiting biofilm formation by up to 82% at 1.6 mg/mL, representing a complementary nano-antimicrobial strategy [165].

Upon absorbing light, the photosensitizer (PS) is excited from its ground state (S_0_) to a short-lived singlet state (S_1_), which then undergoes intersystem crossing to a long-lived triplet state (T_1_), the critical species responsible for generating cytotoxic singlet oxygen (^1^O_2_) [166]. In the subsequent Type II pathway, this triplet PS transfers energy to ground-state molecular oxygen (^3^O_2_) to produce ^1^O_2_ [167]. In Type I processes, the excited photosensitizer instead undergoes electron or proton transfer, producing radical intermediates such as the photosensitizer radical anion and molecular oxygen, which yield superoxide anion (O_2_·^−^), or the photosensitizer radical cation combined with biological electron donors, ultimately generating hydrogen peroxide (H_2_O_2_) and, via Fenton chemistry with Fe^2+^ plus H_2_O_2_, the highly reactive hydroxyl radical (·OH) along with Fe^3+^ and OH^−^ [168].

### 6.4. Quorum-Sensing Inhibition Strategies

Quorum-sensing inhibition (QSI) offers a non-lethal strategy to disarm bacterial pathogens without overt selective pressure. AHL-lactonase Est816 reduces biofilm biomass by 64% and thickness by 76% [169]. Ligand binding triggers LuxR dimerization and DNA association; most LuxR-type receptors use a mechanism in which they dimerize and associate with DNA only upon ligand binding [170]. Despite its appeal as a non-lethal antivirulence strategy, quorum-sensing inhibition faces translational barriers, including redundancy in signaling circuits, strain-specific differences, limited in vivo validation, and uncertain clinical endpoints.

In Gram-positive bacteria, cyclic or linear oligopeptide autoinducers are secreted into the extracellular milieu and detected by membrane-embedded AgrC-type histidine kinase sensors, which then initiate autophosphorylation and phosphotransfer cascades that regulate virulence and biofilm pathways [171]. Interspecies signaling is primarily mediated by the furanosyl borate diester autoinducer-2 (AI-2), a molecule that adopts context-dependent conformations (S-THMF-borate or R-THMF). This universal signal is detected by diverse receptor families, such as the LuxP-LuxQ complex in *Vibrio* species and the LsrB transporter in *E. coli* [172].

The pathogenicity of *P. aeruginosa* is driven by a hierarchical QS network comprising the Las, Rhl, and Pqs systems. These QS circuits coordinately regulate the production of key virulence factors, including exotoxin A, elastase, pyocyanin, rhamnolipids, and siderophores, while simultaneously upregulating biofilm matrix components such as alginate. This integrated regulation of virulence and biofilm formation enables *P. aeruginosa* to evade host defenses and establish persistent infections [173].

QSI operates through three principal mechanisms. First, quorum-quenching enzymes degrade signaling molecules. The endophytic bacterium *Bacillus cereus* AL1 carries the *aiiA* gene encoding an N-acyl homoserine lactonase enzyme that degrades the QS signal molecule N-hexanoyl-L-homoserine lactone (C6-HSL). Nine isolates showed complete degradation (100%) of this signaling molecule, and the partially purified lactonase from *B. cereus* AL1 significantly reduced biofilm formation, swarming motility, and pyocyanin production against *P. aeruginosa* PAO1 and clinical isolates without exerting selective pressure for resistance [174]. Downstream inhibition is equally effective, with natural products like echinacoside reducing intracellular cyclic di-GMP (c-di-GMP) levels and potentiating antibiotics like tobramycin against *P. aeruginosa* aggregates in over 80% of tested strains [175]. A complementary strategy uses oxidoreductases like BpiB09, an NADP-dependent short-chain dehydrogenase/reductase (SDR) that inactivates the key AHL 3-oxo-C12-HSL, resulting in markedly reduced pyocyanin production, motility, biofilm formation, and *Caenorhabditis elegans* paralysis in *P. aeruginosa* [176]. Enzymes such as BpiB09, which are inactivate key AHL signals (e.g., 3-oxo-C12-HSL) through oxidative modification, thereby preventing LuxR-type receptor activation and downstream virulence-gene expression. Unlike bactericidal antibiotics, which impose strong selection for resistance mutations, this enzymatic degradation of signaling molecules disarms pathogens without directly killing them, potentially reducing the selective pressure for classical resistance mechanisms [176,177].

Furanone C-30 strongly binds the LasR QS receptor yet is unable to form the specific interactions needed for the protein’s correct folding and activation. In fact, this compound inhibits the related RhlR receptor independently of LasR, indicating a more complex multi-target mechanism [178]. Recent studies have also identified novel QSI, including cyanophycocyanin, which attenuates *P. aeruginosa* virulence in vivo, and quercetin delivered via star-shaped antibacterial peptide polymers, which downregulate *lasI*, *lasR*, *rhlR*, and *rhlI* to eliminate over 99.99% of biofilms in a corneal keratitis mouse model [179]. The targeting of diguanylate cyclase (DGC) is a promising novel antivirulence strategy to combat AMR. DGC is a multisubunit enzyme that synthesizes c-di-GMP, a key regulator of biofilm formation, motility, and virulence factor synthesis [180]. Recent advances focus on small-molecule and peptide-based therapeutics that inhibit DGC activity, and combining DGC inhibitors with other antibiotics holds significant potential for enhancing treatment efficacy [180].

Taken together, these analyses suggest that the key bottleneck is not only scientific feasibility but also the absence of a development pathway that reliably links clinical evidence, regulatory approval, and a sustainable market for novel antibacterial interventions [122].

### 6.5. Engineered Bacterial Platforms

Engineered commensal bacteria represent another emerging platform for localized delivery of antimicrobial and anti-virulence molecules. For example, probiotic Escherichia coli Nissle 1917 has been engineered to display surface-anchored nanobodies and therapeutic proteins, enabling targeted neutralization of pathogens and toxins in the gut [181,182]. Modified flagellar type III secretion systems have also been exploited to secrete recombinant antimicrobial or anti-virulence proteins from live bacterial carriers, illustrating how synthetic biology can augment traditional small-molecule antibiotics [183].

### 6.6. Vaccines, Monoclonal Antibodies, and Microbiome-Based Approaches

In addition to direct antimicrobial agents, immunological and microbiome-based strategies are increasingly viewed as essential components of a post-antibiotic armamentarium. Conjugate and protein-based vaccines against key pathogens such as *Streptococcus pneumoniae*, *Neisseria meningitidis*, and *Salmonella Typhi* have already reduced the burden of invasive disease and associated antibiotic use [184], and next-generation candidates targeting *Klebsiella pneumoniae*, *Pseudomonas aeruginosa*, and *Clostridioides* difficile are in various stages of development [185]. Monoclonal antibodies and antibody-derived fragments that neutralize bacterial toxins or opsonize encapsulated bacteria offer pathogen-specific protection without broad microbiome disruption [186]. Microbiome modulators, including defined bacterial consortia and fecal microbiota transplantation, aim to restore colonization resistance against multidrug-resistant organisms and reduce recurrent infections [187]. Finally, rational combination therapies that pair traditional antibiotics with adjuvants such as β-lactamase inhibitors, efflux-pump blockers, or quorum-sensing inhibitors seek to extend the useful lifespan of existing drugs and suppress the emergence of resistance.

## 7. The Innovation Void: Why the Pipeline Is Dry

### 7.1. Market Failure

The impending transition to a pre-antibiotic era is being rapidly accelerated by a fundamental and systemic failure of the pharmaceutical market. Too few companies develop new antibiotics because the market fails to deliver sufficient returns on investment. A solution is a delinked payment system that compensates manufacturers based on the societal insurance value of antibiotics, not on sales volume, thereby aligning economic incentives with antimicrobial stewardship and reflecting the direction of current policy reforms [188]. Policy groups have analyzed this market failure for more than a decade. DRIVE-AB and the UK AMR Review both concluded that sales-based antibiotic markets systematically underreward stewardship-compatible products and therefore require delinked or subscription-style incentives. More recently, the UK subscription pilot tested the principle of paying for access value rather than volume, while the UN General Assembly High-Level Meeting on AMR reaffirmed the need for coordinated financing, access, and stewardship reforms. These initiatives show that the market failure problem is not theoretical; it has already become the basis for real policy experimentation.

The antibiotic market is fundamentally broken: economic disincentives have driven major pharmaceutical companies and researchers out of the field, leaving a dangerously thin pipeline that cannot keep pace with rising resistance [189]. Addressing this requires urgent implementation of new models that decouple profit from sales volume, including subscription-style payments, push-and-pull incentives, and public–private partnerships such as CARB-X and GARDP [189]. Companies both active and prospective in antibiotic development confront major economic hurdles, coupled with a limited and unpredictable future market. Analyses of antibiotic R&D economics have produced different rNPV estimates because they use different assumptions regarding development costs, discount rates, probability of technical and regulatory success, market size, and projected revenues. One model estimated the rNPV of a typical antibiotic project at approximately -US$50 million, whereas another analysis reported an rNPV of approximately US$100 million. These values are therefore not directly interchangeable. Despite this methodological variation, both estimates are substantially lower than the approximately US$1.15 billion reported for musculoskeletal drugs, supporting the conclusion that antibiotic development remains economically unattractive without delinked incentives [190]. Antibiotic development is economically unsustainable: bringing a new drug to market costs $1.2 billion, yet stewardship limits annual sales to $100 million, a tenfold difference [191]. This work is scientifically essential but commercially unrewarding, a mismatch that has driven antibacterials to capture <5% of venture capital, while FDA approvals have fallen from 20% (1980) to 6% today [191].

Companies developing antibiotics are failing despite the pressing global threat of AMR. To address this, large pharmaceutical companies created the AMR Action Fund, which will invest $1 billion in small antibiotic development companies to bring 2–4 new agents to the clinic by 2030 [192]. However, while the Fund provides an immediate lifeline to companies unable to secure investment, it will not fix the underlying broken development model. Ultimately, the Fund buys time for essential market reforms to salvage antibiotic development [192].

A novel framework for quantifying public investment in antibiotic research and development is piloted, analyzing 56 compounds developed largely by small-to-medium-sized enterprises (SMEs). Public research funding is the most frequently cited source of support for SMEs across both early and late development stages [193]. A case study of the antibiotic Venatorx reveals that public contributions, approximately $655 million, substantially exceeded private investment, highlighting the magnitude of public sector involvement in this field. The framework is deemed feasible and could inform pharmaceutical price negotiations by accounting for prior public funding [193].

The collapse of Achaogen serves as a chilling case study of this market failure. The company successfully developed and commercialized plazomicin, a novel aminoglycoside approved by the FDA in 2018 for treating complicated urinary tract infections caused by multidrug-resistant *Enterobacterales* [194]. Despite this regulatory success, Achaogen was unable to fund the drug’s commercialization and filed for bankruptcy just ten months after launch, revealing that for small biotechnology firms, inventing a needed antibiotic is no longer enough to survive [195]. The lack of commercial success for newly launched antimicrobial agents provides little incentive to invest in the development of new agents. For example, Achaogen, a company that filed for bankruptcy in April 2019, launched plazomicin in July 2018 and reported just $800 000 in total sales for 2018 before filing for bankruptcy [194]. This reality underscores a core market failure: even a clinically useful new drug could not generate enough revenue to cover its development and post-marketing costs [194].

Most non-traditional products can still be developed within existing regulatory frameworks using non-inferiority or superiority trial designs, but products with primarily population-level goals may require extensions of current paradigms to capture their full public-health value [122].

### 7.2. Pipeline Disparity

The search for new β-lactamase inhibitors is critical, yet the current pipeline of novel β-lactam antibiotics remains alarmingly sparse. A major disparity exists: clinically approved inhibitors are ineffective against MBLEs, the most concerning class of resistance enzymes [196]. The situation is exacerbated by the increasing prevalence of carbapenemase-producing bacteria, particularly those carrying the highly adaptable NDM-1 enzyme, for which no clinically approved compound exists [196]. The antibiotic pipeline remains critically weak, with most candidates being incremental modifications of existing drug classes rather than novel agents. This disparity is especially pronounced for resistant Gram-positive pathogens such as VRE and daptomycin-nonsusceptible *S. aureus*, where new treatment options are virtually absent [197].

Since 2010, only a limited number of new antibiotic classes have been approved, underscoring a serious innovation gap [198]. Unregulated antibiotic use and weak surveillance systems, particularly in LMICs, further accelerate resistance spread. AMR is an escalating, measurable crisis that is undermining decades of progress in infectious disease control, and urgent incentives are needed to revitalize antibiotic R&D [198].

### 7.3. The Diagnostics Gap

Resistance mechanisms are evolving faster than new drugs can be developed, putting many existing antibiotics at risk of becoming ineffective. Addressing AMR demands a multi-faceted strategy that includes funding for novel antimicrobials, strengthening global surveillance systems, and implementing rigorous infection prevention and control measures [198]. For nearly a century, the clinical gold standard for identifying bacterial infections has remained culture and antimicrobial susceptibility testing (AST). While phenotypic AST provides definitive data, it is inherently limited by the biological growth rates of pathogens, typically requiring 48 to 72 h to yield actionable results [199]. A Malaysian ICU study found that 48.7% of patients received appropriate antibiotics within the recommended time window, whereas overall compliance with the broader antibiotic-administration protocol was 51.3%. The small gap between these percentages reflects patients who met some but not all protocol criteria, rather than contradictory outcomes [200]. Overall survival was just 60.3%, and delays beyond one hour significantly increased mortality [200]. These data highlight a critical diagnostic gap where non-specific early symptoms, limited access to rapid tests, and slow blood culture results push patients past the one-hour window, reducing survival.

The rise in AMR and neglected infectious diseases demands rapid, decentralized diagnostic solutions. Delays in accurate diagnosis, especially in low-resource settings, lead to inappropriate antibiotic use and fuel resistance. Point-of-care molecular diagnostics, such as isothermal amplification and CRISPR-based platforms, can provide results within 15–60 min, enabling early resistance profiling and guiding appropriate therapy at the first clinical encounter [201]. Without such technology, antibiotic stewardship remains largely aspirational, limited by the lag time of traditional microbiology. Promising technological innovations have emerged, including CRISPR-Cas-based diagnostic platforms that detect specific nucleic acid sequences with high sensitivity. These programmable systems leverage the collateral cleavage activity of Class II Cas effectors to achieve sensitivity and single-nucleotide specificity, enabling rapid identification of resistance markers directly from clinical samples without prolonged culture steps [202]. Emerging next-generation lateral flow assays have evolved to detect bacterial and fungal pathogens with improved sensitivity and speed, offering a promising solution to bridge the diagnostics gap in resource-limited settings [203]. Current diagnostics are largely confined to centralized laboratories, leaving major gaps at primary care levels. No simple, affordable, point-of-care solution yet exists for reliable pathogen identification and resistance profiling. These limitations are especially acute in low-resource settings, where consistent power, trained staff, and supply chains are lacking. A shift toward robust, low-cost, user-friendly diagnostic tools is urgently needed [204].

### 7.4. Non-Traditional Therapies

Bacteriophages can eradicate *P. aeruginosa* biofilms by degrading the extracellular matrix, enhancing antibiotic penetration into deep biofilm layers, and suppressing QS-mediated biofilm formation [205]. Despite challenges such as narrow host range and emergence of resistant subpopulations, phage therapy holds promise for treating chronic, antibiotic-resistant *P. aeruginosa* infections [205]. Phage cocktails evade resistance through synergistic lytic cycles and can be rapidly evolved against emerging strains, offering precision antimicrobial action unattainable by broad-spectrum drugs [206].

Antimicrobial peptides (AMPs), including colistin alternatives and synthetic cathelicidin derivatives, act through multi-target mechanisms involving both membrane disruption and intracellular interference, which collectively reduce the likelihood of resistance emergence. In contrast to conventional single-target antibiotics, AMPs rely on rapid, primarily physical membrane perturbation, resulting in a much lower incidence of resistance development [207]. Host defense peptides interact with pattern recognition receptors to modulate cytokine production, recruit immune cells, and regulate inflammation, enabling effective pathogen clearance while minimizing disruption of the host microbiome, a key advantage over conventional broad-spectrum antibiotics [208]. A 2026 study on LysSW21, an endolysin derived from a *Staphylococcus* phage, demonstrated potent activity against MRSA. The enzyme showed MICs of 12.5–25 μg/mL, and at MIC levels it reduced bacterial viability by 4–5 log_10_ units, achieving complete eradication of planktonic MRSA within 75–105 min [209]. This rapid killing kinetics is orders of magnitude faster than conventional antibiotics, which typically require hours to achieve similar reductions. LysSW21 also retained high stability across a broad range of temperatures and pH values, disrupted 70–80% of pre-formed biofilms, and significantly reduced biofilm formation under static conditions [209]. The engineered endolysin LNT103 exhibited a synergistic effect with colistin and additive effects with carbapenem antibiotics. In vivo, it led to the re-sensitization of resistant bacteria to meropenem [210]. Similarly, the endolysin LysAB1245, when combined with colistin, did not confer bacterial resistance after multiple passages. The synergistic bactericidal effect, achieving a >3-log reduction in bacterial counts within 4 h, was demonstrated without selecting for resistant mutants. [211].

Realizing the full potential of these non-traditional therapies will require not only sustained scientific investment but also adaptive regulatory pathways and new economic models that recognize their unique value in preserving the effectiveness of both novel and existing antibiotics.

## 8. Global Governance and the Implementation Gap

### 8.1. The Mirage of Action

Global AMR governance scores rose from 30.7 to 44.5 (out of 100) between 2017 and 2022, yet meaningful reductions in resistance only appeared around five years after a National Action Plan’s (NAP) adoption, with multi-sector engagement and robust antimicrobial-use surveillance being the primary drivers [212]. The persistent failure to translate political commitments into measurable reductions in AMR stems from an implementation gap driven by weak metrics, misaligned priorities, and structural inequalities. Addressing this gap requires a structural antibiotic policy that targets economic, political, and cultural root causes, Equitable that involves fairly distributing stewardship burdens and access benefits, and tracked, which uses transparent monitoring to ensure accountability and adaptive learning [213]. Without these three hallmarks, NAPs risk becoming performative documents rather than instruments of change [213].

The 2015 adoption of the Global Action Plan (GAP) on AMR was indeed a diplomatic success that elevated AMR to the top tier of global health priorities [214]. Despite political endorsements, a notable gap between official commitments and on-the-ground execution persists. By 2023, while 178 nations had formulated NAPs for AMR, actual implementation has been described as alarmingly fragmented. Specifically, only 27% of these plans show operational effectiveness, and just 11% benefit from dedicated national funding [214]. Challenges span One Health sectors, including a lack of ring-fenced national funding for NAP implementation and weak coordination both across ministries and within them. This has resulted in what can be described as a largely symbolic policy environment, where high-level strategies exist only on paper and are poorly executed, consequently failing to tackle the fundamental drivers of AMR [215].

The decreasing effectiveness of antibiotics has quickened in recent years, and with the arrival of untreatable strains of carbapenem-resistant Enterobacteriaceae, we are at the dawn of a post-antibiotic era [216]. In LMICs, antibiotic use is increasing with rising incomes, high rates of hospitalization, and high prevalence of hospital infections. Modern medicine, major surgery, organ transplantation, treatment of preterm babies, and cancer chemotherapy would not be possible without effective antibiotics. Within just a few years, we might be faced with dire setbacks, medically, socially, and economically, unless real and unprecedented global coordinated actions are immediately taken [216].

Policy measures to restrict non-prescription antibiotic sales often remain symbolic. Research demonstrates that in many low-resource settings, regulations are weakly enforced because strict prescription-only laws would cut off access to essential medicines for rural populations, creating an implementation gap that perpetuates antibiotic misuse [217]. Antimicrobial stewardship in tertiary care hospitals is consistently obstructed by multilevel barriers, including gaps in healthcare professional knowledge, chronic shortages of staff and resources, and systemic governance failures. These obstacles are especially severe in LMICs, where formal action plans often remain unimplemented due to a lack of foundational infrastructure and workforce capacity [218].

### 8.2. The Post-COVID Context

The COVID-19 pandemic exposed how fragile antimicrobial stewardship can be globally, and it significantly accelerated the AMR crisis. Early in the pandemic, clinical uncertainty caused routine evidence-based prescribing to break down at an enormous scale. A 2023 meta-analysis by Calderon and colleagues synthesized data from more than 30,000 hospitalized patients and put numbers to this failure: although only 4% had confirmed bacterial co-infection, antibiotic administration reached 60% [219]. Globally, the COVID-19 pandemic triggered a marked surge in unnecessary antibiotic use. An analysis synthesizing 29 studies across 11 nations, encompassing more than 150,000 hospitalized patients, reported that antibiotics were administered to 67% of individuals admitted with COVID-19 [220].

During 2020-2021, COVID-19-associated mucormycosis turned a previously rare fungal disease into a near-epidemic in India, with over 30,000 recorded cases [221]. Two major gaps became clear: a lack of rapid diagnostic tools and the failure of amphotericin B once the infection reaches necrotizing tissues, leaving surgery as the only option. Uncontrolled diabetes and cancer were linked to the highest death rates. This fungal co-epidemic mirrors the broader AMR crisis, delayed diagnosis, last-line drug failure, and a heavy toll on vulnerable groups, highlighting the urgent need to integrate fungal threats into global resistance surveillance frameworks [221].

The pandemic placed immense strain on global health surveillance systems, extending well beyond the direct clinical toll. Overwhelmed healthcare infrastructures resulted in what has been described as the diversion of health system resources to the pandemic response. Consequently, AMR surveillance and stewardship programs were de-emphasized, delayed, or halted [222].

## 9. Discussion: Are We Past the Point of No Return?

The narrative of a pre-antibiotic era has shifted from a hyperbolic warning to a data-driven inevitability that is already shaping clinical outcomes. We are currently witnessing the bifurcation of global health into two distinct realities. For many people in LMICs, the threat of untreatable infections is already a reality. First-line treatments, like the ample use of ampicillin, are frequently ineffective, as a systematic review and meta-analysis of community-acquired enteric pathogens among young children showed a pooled resistance rate of 56% for *E. coli* against ampicillin. The effectiveness of ciprofloxacin, another widely used oral agent, is also seriously compromised, with a pooled resistance rate of 10% in the same *E. coli* isolates [223]. This gap in access, driven by weak health systems and poor implementation of NAPs, means that many people with highly drug-resistant infections are not getting the treatment they need, resulting in increased morbidity and mortality [224].

Current global strategies to bridge this divide rely heavily on soft governance mechanisms. These include voluntary NAPs, public awareness campaigns, and non-binding political commitments made at high-level summits. Conjugative plasmids play a critical role in the global pandemic of AMR by enabling the horizontal transfer of resistance genes at rates that can rapidly outpace many traditional intervention strategies [225]. To avert a permanent regression in medical capability, the global response must shift from voluntary stewardship to mandatory, enforceable regulation, as seen in China’s nationwide mandatory antimicrobial stewardship program [226]. In 2022, the UK’s National Health Service introduced the world’s first subscription-type payment model for ceftazidime-avibactam and cefiderocol, and a 2025 survey of antimicrobial stewardship pharmacists found strong support for the model, with 88.4% endorsing its principle of promoting stewardship and innovation [227].

### 9.1. The Role of Surveillance

A 2025 analysis of six countries found that while all have established national surveillance systems for AMR, implementation is profoundly uneven: progress on key steps for sustainable NAPs ranged from just 7% in Burundi to 94% in Kenya, while WHO surveillance checklist scores varied from 44% in South Sudan to 100% in Uganda and Kenya [228]. These disparities reflect persistent systemic weaknesses, limited laboratory testing capacity, non-representative data, fragmented cross-sectoral reporting, and chronic underfunding, which collectively undermine efforts to obtain reliable AMR estimates [228].

Placing a surveillance system in the spotlight often reveals as much about its weaknesses as its strengths. The WHO’s GLASS-AMR initiative, designed to unify national surveillance systems to estimate the global scale and burden of AMR, is a case in point [229]. A closer look at its implementation shows that while all countries submitted data by 2021, representation and data quality varied substantially between them, driven by complex data systems and gaps in laboratory quality assurance [229]. Ultimately, these data sensitivity, representativeness, and timeliness issues seriously compromised the system’s ability to guide timely local action, underscoring that effective surveillance must first be sustainable and generate actionable, high-quality data on the ground [229].

Complementing the WHO GLASS and regional NAP assessments discussed above, the CDC’s most recent national threats report documents a persistent gap between surveillance coverage and actionable, near-real-time resistance data, particularly for carbapenem-resistant *Enterobacterales* and *Acinetobacter* species. Aligning GLASS indicators with faster national laboratory turnaround could shorten the interval between pathogen detection and stewardship response, echoing this review’s diagnostics-gap argument in Section 6.3. Incorporating this cross-agency comparison would reinforce the manuscript’s claim that sustained surveillance investment, not diagnostic innovation alone, remains the binding constraint on equity-focused AMR containment [230,231].

Genomic surveillance offers a transformative solution to these limitations. Unlike phenotypic testing, whole-genome sequencing can track specific resistance genes, MGEs, and transmission networks in real time across borders, enabling high-resolution outbreak tracing and AMR-gene mapping [232]. Despite its potential, genomic surveillance remains largely confined to academic research projects and specialized networks rather than being integrated into routine public health decision-making. However, the barrier has shifted from the cost of generating data to the complexity of analyzing it, as large-scale sequence data requires robust bioinformatics pipelines, storage, and interpretive capacity [233].

### 9.2. Equity and Access

The crisis of AMR is fundamentally a crisis of equity. AMR disproportionately impacts LMICs, accelerating health inequities and creating a two-tier world of treatable and untreatable infections [234]. The burden of resistant infections falls disproportionately on the populations with the fewest resources to defend themselves, where infection-prevention capacity, diagnostic infrastructure, and access to high-quality antimicrobials are weakest [235]. Neonates born in low-resource hospitals, immunocompromised patients in sub-Saharan Africa and South Asia, and surgical patients in regions without access to clean water and sanitation face the highest risks of acquiring untreatable infections, as surveillance and stewardship gaps drive high rates of MDR pathogens in these settings [236].

In Southeast Asia, neonatal AMR data remain sparse, but where microbiology capacity exists, resistance rates are extreme. Adjusted pooled non-susceptibility of *Klebsiella* spp. reached 86.7% for third-generation cephalosporins and 17.1% for carbapenems; for *Acinetobacter* spp., carbapenem non-susceptibility was 76.5%. [236]. Overcrowding, limited staffing, and laboratory supply shortages at multiple sites worsen these outcomes. Even when neonates reach tertiary hospitals, empirical treatment fails at staggering rates, reflecting deep inequities in access to effective infection care [236].

## 10. Limitations, Translational Challenges and Research Priorities

Although the alternative strategies reviewed here show considerable promise, important limitations constrain their immediate clinical impact. Phage therapy is hindered by narrow host ranges, the rapid emergence of phage-resistant bacterial subpopulations, and potential immune clearance of phage particles, necessitating personalized cocktails and careful patient selection [237,238]. Antimicrobial peptides face challenges related to proteolytic instability, potential host cytotoxicity, high manufacturing costs, and variable pharmacokinetics in complex infection sites [239,240]. Photodynamic therapy is limited by light penetration depth, the need for local delivery of photosensitizers, and variable efficacy in deep-tissue or disseminated infections. Enzyme-based therapies, including endolysins and depolymerases, must overcome immunogenicity, rapid systemic clearance, and difficulties in reaching intracellular or biofilm-embedded pathogens [241]. CRISPR-Cas-based antimicrobials, despite their sequence-specific targeting potential against resistance genes or pathogenic bacteria, are similarly constrained by inadequate in vivo delivery efficiency, off-target cleavage risks, and bacterial escape through target-site mutations or anti-CRISPR proteins [242,243]. For all non-traditional modalities, scalable manufacturing, robust quality control, and clear regulatory pathways remain unresolved in many jurisdictions [191]. Finally, most evidence to date derives from in vitro or animal models; well-designed clinical trials with standardized endpoints are urgently needed to establish efficacy, safety, and optimal use in diverse patient populations [244,245].

Several critical knowledge gaps must be addressed to translate promising concepts into routine clinical practice. Priority areas include: (i) the design of adaptive clinical trials for phage and enzyme therapies that accommodate strain specificity and evolving resistance [246]; (ii) the development of formulation and delivery systems that protect antimicrobial peptides and enzymes from degradation while enabling targeted release at infection sites [247]; (iii) the systematic mapping of resistomes across human, animal, and environmental compartments to identify high-risk transmission nodes and inform One Health interventions [248,249]; (iv) the discovery and optimization of small-molecule adjuvants that inhibit β-lactamases, efflux pumps, or virulence circuits without imposing strong selection for resistance [250]; (v) the integration of rapid point-of-care diagnostics with real-time genomic surveillance to guide pathogen-specific therapy and stewardship decisions in both high-resource and low-resource settings [251]; and (vi) the integration of artificial intelligence and machine learning into different stages of the discovery and optimization process could streamline development, from predicting peptide structures and phage-host interactions to refining photosensitizer properties, while reducing both time and cost [252]. Addressing these priorities will require sustained interdisciplinary collaboration among microbiologists, clinicians, engineers, data scientists, and policymakers.

## 11. Future Directions and Conclusion

Robust surveillance infrastructure forms the indispensable foundation for AMR containment, yet persistent gaps, especially in LMICs, severely limit global response capacity. The WHO GLASS 2025 report reveals that only 40% of reporting countries achieve near-real-time data integration, with many LMICs facing sub-50% laboratory coverage for WHO-defined critical-priority pathogens like CRAB and CRKP. Consistent with the uneven NAP implementation reported in East Africa, many LMICs still lack integrated human–animal–environmental surveillance and adequate laboratory coverage for critical-priority pathogens. Achieving 80% national laboratory capacity by 2030 demands coordinated investment in standardized AST, genomic sequencing, and linked antimicrobial-use metrics across One Health sectors to enable predictive modeling of resistance waves and preemptive stewardship.

True One Health demands integrated human–animal–environmental surveillance over silos. Assessments confirm LMICs often lack these links, with regulatory gaps in pharmaceutical effluents and agricultural use fueling environmental reservoirs. Wastewater resistome monitoring and triad platforms provide early warning for threats like *mcr-1* spread from farms. Equity-focused strategies avert a two-tier post-antibiotic world: neonatal-sepsis protocols for ESBL/CRAB (e.g., Southeast Asia’s 86% cephalosporin resistance in Klebsiella), WHO treatment packages, community stewardship training, regional labs, and behavioral interventions tailored for LMICs like India.

The transition to a pre-antibiotic era is no longer theoretical but an unfolding crisis eroding modern medicine’s foundations. Surgeries, chemotherapy, transplants, and childbirth now risk untreatable complications. This stems from biological-evolutionary drivers (antibiotic overuse, HGT, adaptability) clashing with economic short-termism. Moving beyond voluntary NAPs (only 27/178 operational) requires enforceable actions.

These four pillars, including comprehensive surveillance, diversified innovation (phages, AMPs, PDT), integrated One Health governance, and equity programming- offer escape routes. Economic reforms like subscription models (e.g., the UK’s ceftazidime-avibactam pull-incentive, endorsed by 88% of pharmacists) delink payments from volume to revive pipelines (rNPV $100M vs. $1.15B needed). Global frameworks should restrict non-therapeutic antimicrobial use in agriculture. Expand labs with CRISPR diagnostics (15-60 min results) and local manufacturing for affordability. Without binding 2030 targets, the post-antibiotic reality arrives in a decade. Scientific innovation alone will not resolve AMR unless it is matched by financing reform, equitable access, stewardship enforcement, and stronger global governance.

## Figures and Tables

**Table 1:** The ESKAPE Pathogens and the Threat of a Pre-Antibiotic Era.

Pathogen	Key Resistance Mechanisms	Clinical Implications in a Pre-Antibiotic Era	Ref.
*Enterococcus faecium*	Vancomycin resistance	Loss of standard therapy for endocarditis and catheter-associated infections.	[30]
*Staphylococcus aureus*	Methicillin resistance; vancomycin-intermediate/resistance; biofilm formation	Compromised safety for orthopedic joint replacements and cardiothoracic surgery.	[31,32]
*Klebsiella pneumoniae*	Colistin-resistant, carbapenemase-producing	High-mortality outbreak across NICU, ICU (Intensive Care Unit), and wards; containment failure despite enhanced infection control	[33]
*Acinetobacter baumannii*	Carbapenem resistance, Pan-drug resistance	High-mortality ICU pathogen	[34]
*Pseudomonas aeruginosa*	Carbapenem resistance, multidrug efflux pumps	Higher mortality and graft failure in solid organ transplant recipients	[35]
*Enterobacter* species	Carbapenem resistance, Extended-Spectrum Beta-Lactamase production, AmpC β-lactamases	Untreatable healthcare-associated infections (bloodstream, ventilator-associated pneumonia, UTIs, surgical-site infections)	[36]

**Table 2:** The Triad of Resistance: One Health Drivers and Transmission Pathways.

Domain	Primary Drivers	Key Resistance Elements	Transmission Pathway to Humans	Ref.
Human Health	Poor healthcare infrastructure, weak antimicrobial policies and stewardship, and poor sanitation	ESBL (Extended-spectrum β-lactamase) producing *Klebsiella pneumoniae* and *Escherichia coli*; carbapenem-resistant *Enterobacterales*	Nosocomial; household; livestock contact; international travel	[61]
Agriculture and Food	Antimicrobial use in livestock and humans; intensive poultry farming practices	*blaNDM*, *blaKPC*, *blaOXA-48-like*	Consumption of contaminated raw chicken meat; potential environmental spread through animal–human contact	[62,63]
Environment	Pharmaceutical effluent; improper waste disposal	Antibiotic-resistant bacteria (ABRB) and resistance genes (ARGs) selected for in the environment	Contaminated water sources; bioaccumulation through the food chain	[64]

**Table 3:** Mechanistic and Therapeutic Profiles of Phage-based therapy and Antimicrobial Enzymes.

Therapy	Mechanism	Applications	Challenges	Ref.
Phages	Viruses that infect and lyse bacterial cells; disrupt biofilm matrix via depolymerases	Treatment of antibiotic-resistant infections; biofilm elimination; adjunct to antibiotics	Narrow host range; bacterial resistance; immune clearance; regulatory/manufacturing hurdles	[129,130,131]
Lysostaphin	Zn^2+^-dependent metalloendopeptidase that lyses *S. aureus* by cleaving Gly-Gly bonds in the pentaglycine cross-bridges of its cell wall	Treating implant-associated MRSA infections	Clinical use is limited by poor shelf stability and limited bioavailability	[132,133,134]
Glucose oxidase	It catalyzes β-D-glucose to gluconic acid and H_2_O_2_ using FAD cofactor and molecular oxygen as electron acceptor	GOx generates H_2_O_2_ for antimicrobial action and gluconic acid to lower pH in hyperglycemic wounds	Rapid proteolytic degradation, short plasma half-life, off-target toxicity limit clinical translation	[135,136,137]
Dispersin B	hydrolyzes β-1,6-N-acetyl-D-glucosamine linkages in PNAG biofilm matrix	Chronic wound treatment - disperses biofilms to enhance antimicrobial penetration in diabetic ulcers	Limited clinical translation due to rapid enzymatic clearance, proteolytic degradation, and poor tissue penetration	[138,139,140]

**Table 4:** Molecular Mechanisms and Translational Potential of AMPs.

AMP Type/Example	Mechanism	Key Feature	Limitation	Ref.
Natural Broad (LL-37)	Membrane disruption (carpet/toroidal pores) and immunomodulation	Kills > 38 bacteria, 16 fungi, and 16 viruses; Phase II trial for venous leg ulcers	Susceptible to protease degradation and cytotoxicity; high production costs	[148,149,150]
Natural Targeted (Nisin)	Binds to Lipid II, which inhibits cell wall synthesis and also forms pores in the bacterial membrane	Holds GRAS status and is FDA-approved as a food preservative	Ineffective against Gram-negative bacteria due to the outer membrane barrier. Also susceptible to protease degradation	[151,152,153]
Synthetic Broad (SAAP-148)	Destroys bacterial membranes through a carpet-like mechanism rather than forming discrete pores	Kills MDR pathogens, biofilms, and persisters without resistance; 4 h topical treatment clears MRSA from mouse skin	Cytotoxicity and short half-life; C-terminal PEGylation reduces hemolysis and enhances immunomodulation but adds formulation complexity.	[154,155,156]
Synthetic Targeted (WR12)	Forms an amphipathic α-helix that kills bacteria by permeabilizing their membrane	Eradicates MRSA persisters and established biofilms; topical application in mice reduces bacterial load and pro-inflammatory cytokines in skin lesions.	None reported; further in vivo toxicological and pharmacokinetic studies are needed.	[157,158,159]

## Data Availability

No new data were created or analyzed in this study. Data sharing is not applicable to this article.

## Abbreviations

ABC	ATP-binding cassette
AHLs	N-acyl homoserine lactones
AMEs	Aminoglycoside-modifying enzymes
AMPs	Antimicrobial peptides
AMR	Antimicrobial resistance
ARGs	Antibiotic resistance genes
AST	Antimicrobial susceptibility testing
c-di-GMP	Cyclic di-GMP
CRAB	Carbapenem resistance *Acinetobacter baumannii*
CRKP	Carbapenem-resistant *Klebsiella pneumoniae*
CRPA	Carbapenem-resistant *Pseudomonas aeruginosa*
DGC	Diguanylate cyclase
eDNA	Extracellular DNA
EMA	European Medicines Agency
EPS	Extracellular polymeric substance
ESBL	Extended-spectrum β-lactamase
FDA	Food and Drug Administration
GARDP	Global antibiotic research and development partnership
GDP	Gross domestic product
GlcNAc	N-acetylglucosamine
GRAM	Global research on antimicrobial resistance
HGT	Horizontal gene transfer
ICU	Intensive care unit
LMICs	Low- and middle-income countries
LPS	Lipopolysaccharide
MBLE	Metallo-β-lactamase
MDR	Multidrug-resistant
MGEs	Mobile genetic elements
MICs	Minimum inhibitory concentrations
MRSA	Methicillin-resistant *Staphylococcus aureus*
MurNAc	Β-1,4-N-acetylmuramic acid
NAPs	National action plans
NDM-1	New Delhi metallo-beta-lactamase 1
NICU	Neonatal intensive care unit
PDR	Pandrug-resistant
PDT	Photodynamic therapy
PG	Peptidoglycan
PNAG	Poly-β-1,6-N-acetyl-D-glucosamine
QRDRs	Quinolone resistance-determining regions
QS	Quorum sensing
QSI	Quorum-sensing inhibitors
rNPV	Risk-adjusted net present value
ROS	Reactive oxygen species
SMEs	Small-to-medium-sized enterprises
TA	Toxin-antitoxin
VISA	Vancomycin-intermediate *Staphylococcus aureus*
VRE	Vancomycin-resistant *Enterococcus*
VRSA	Vancomycin-resistant *Staphylococcus aureus*
WHO	World Health Organization
XDR	Extensively drug-resistant
